# Origins of the Tumor Microenvironment: Quantitative Assessment of Adipose-Derived and Bone Marrow–Derived Stroma

**DOI:** 10.1371/journal.pone.0030563

**Published:** 2012-02-20

**Authors:** Shannon Kidd, Erika Spaeth, Keri Watson, Jared Burks, Hongbo Lu, Ann Klopp, Michael Andreeff, Frank C. Marini

**Affiliations:** 1 Section of Molecular Hematology and Therapy, Department of Leukemia, M. D. Anderson Cancer Center, The University of Texas, Houston, Texas, United States of America; 2 Department of Radiation Oncology, M. D. Anderson Cancer Center, The University of Texas, Houston, Texas, United States of America; University of Medicine and Dentistry of New Jersey, United States of America

## Abstract

To meet the requirements for rapid tumor growth, a complex array of non-neoplastic cells are recruited to the tumor microenvironment. These cells facilitate tumor development by providing matrices, cytokines, growth factors, as well as vascular networks for nutrient and waste exchange, however their precise origins remain unclear. Through multicolored tissue transplant procedures; we have quantitatively determined the contribution of bone marrow-derived and adipose-derived cells to stromal populations within syngeneic ovarian and breast murine tumors. Our results indicate that subpopulations of tumor-associated fibroblasts (TAFs) are recruited from two distinct sources. The majority of fibroblast specific protein (FSP) positive and fibroblast activation protein (FAP) positive TAFs originate from mesenchymal stem/stromal cells (MSC) located in bone marrow sources, whereas most vascular and fibrovascular stroma (pericytes, α-SMA^+^ myofibroblasts, and endothelial cells) originates from neighboring adipose tissue. These results highlight the capacity for tumors to utilize multiple sources of structural cells in a systematic and discriminative manner.

## Introduction

Constituents of the tumor microenvironment can arise from two major avenues: recruitment from nearby local tissue or systemic recruitment from distant tissue via circulation. Though constitution will vary from tumor to tumor, very little is definitively understood about the composition and origin of the host-derived cellular milieu found within the various tumor microenvironments. The most accessible option for tumor cells engaged in stromal cell recruitment is to exploit resources in close proximity to the site of tumor development. Dependent upon anatomical location, these tissues are often rich sources of fibroblasts, pericytes and vascular cells, as all cell types are critical for normal tissue function as well.

Work by Udagawa et al. investigated the local cellular contribution to the tumor microenvironment by transplanting skin from a ubiquitously expressing green fluorescent protein (GFP)-expressing mouse and establishing tumors in the subcutaneous space beneath the engrafted skin [1]. Their findings suggest most of the tumor CD31^+^ vessels are recruited from cells within the nearby GFP^+^ tissue using either a murine syngeneic lung carcinoma or a xenogeneic osteosarcoma models. Additionally, studies focusing on fibrosis leading to cancer development have identified activated tissue resident cells responsible for excessive extracellular matrix (ECM) production, such as pancreatic stellate cells in pancreatitis that induce progression to pancreatic cancer [2] or peribronchiolar and perivascular adventitial lung fibroblasts that lead from lung fibrosis to lung cancer development [3].

Though not as easily accessible as local tissue, accumulating evidence has been presented suggesting recruitment from more distant cell sources, such as bone marrow. In cases of rapid tumor development, local cells may not be capable or in sufficient numbers to meet expanding growth demands. Additionally, as tumor vascular networks expand, access to systemically circulating cells in the blood supply increases concurrently. Accordingly, many findings have implicated extensive bone marrow contribution to the tumor microenvironment.

Both bone marrow and adipose derived endothelial and mesenchymal progenitor cells have been isolated, cultured and injected back into mice to show that they possess both tumor tropic and tumor promoting capacity [4]–[8]. Furthermore, several studies have addressed the contribution of bone marrow derived cells to the tumor microenvironment utilizing transgenic mouse models [9], and human bone marrow transplant patient tumor samples [10]. The aforementioned studies suggest that bone marrow derived cells contributed to less than 20% of the stroma found in the tumor microenvironment, therefore, in our study, we sought to address the origin(s) of the remaining percentage of tumor associated stroma.

As hematopoietic cells, all immune cells originate from the bone marrow, and the extensive contribution of immune cells in tumors such as macrophages and lymphocytes has been well documented [11]–[13]. In addition, our group recently demonstrated that bone marrow derived mesenchyme contributes to vascular and fibroblastic structures within the tumor microenvironment [7], [14]. Although these results are likely to be dependent on tumor type and experimental conditions, evidence from us and others clearly present several roles for non-immune bone marrow derived cells in the tumor microenvironment. Additional evidence for bone marrow originating circulating populations contributing to tumor stroma is provided in a few studies that report the existence of a circulating bone marrow derived endothelial progenitor cells (EPCs) capable of contributing 10–50% tumor associated endothelial cells in certain animal models [15]–[18]. Next, bone-marrow derived α-SMA+ myofibroblasts have been cited to contribute between 0–30% of stromal isolated fibroblasts within various tumor contexts [9], [19]–[21]. Finally, recent publications have proposed a bone marrow origin for pericytes within the tumor vasculature [22]–[26]. These above example suggest that bone marrow derived cells can contribute to multiple stromal compartments in the tumor microenvironment.

Recruited tumor associated fibroblasts (TAFs) have been identified as central participants in tumor remodeling and structural matrix formation. These cells are often characterized by increased expression of pathology-associated or “activated” fibroblast markers, fibroblast specific protein (FSP) and fibroblast activation protein (FAP); increased expression of markers of aggression and pro-tumorigenic growth factors; and markers of fibrovascularization such as α-smooth muscle actin (α-SMA) and desmin. The origin of TAFs is not well understood, but recent evidence from our lab and others indicate bone marrow derived mesenchymal stem cells (BM-MSC) are a source of TAFs [7], [27]–[29]. BM-MSC have been well characterized for their tropism for inflammatory microenvironments such as wounded tissue and tumors [30]. Within wounded tissue, MSC certainly serve a beneficial role in aiding the healing process, however, the role of MSC within the tumor microenvironment is not quite as clear.

In this investigation, we sought to determine the origins of TAFs and vascular stromal elements in the tumor microenvironment. By performing a series of multi-colored bone marrow and adipose tissue transplantations prior to tumor establishment, we were able to quantitate endogenous contributions to these populations as measured by multiple phenotypic markers, and determine the tissue of origin of these cells. Our results demonstrate that bone marrow mesenchyme, potentially as mesenchymal stem cells, is recruited into tumors as FSP+/FAP+ TAFs, whereas the vascular stroma (pericytes and fibrovascular structures) defined by αSMA+/NG2+ TAFs as well as endothelial cells is recruited from neighboring adipose tissue. These data suggest the recruitment of two distinct subpopulations of TAFs each with a discrete tissue of origin.

## Results

### Relative contribution of bone marrow-derived vs. local resident tissue in ID8 ovarian tumors

In the first set of experiments, bone marrow from GFP expressing mice was transplanted into lethally irradiated RFP mice. Upon engraftment, as evidenced by >95% GFP expression in the peripheral blood and the death of control mice not receiving bone marrow, the mice received ID8 ovarian tumors (Figure S1).

Analysis of tumor sections revealed that GFP^+^ and RFP^+^ stromal cells (of non-tumor origin) comprised 23+/−3% of cells found within the tumor mass. These cells were found both around the periphery and within the parenchyma of the tumor. Of the stromal cells, 42+/−9% were bone marrow-derived and 58+/−6% were non-bone marrow-derived, representing 10+/−2% and 14+/−1% of the tumor bulk, respectively.

Next, we analyzed the tumor sections for phenotypic markers generally associated with tumor associated fibroblasts (TAFs), including αSMA, NG2, FAP and FSP. Interestingly, under our experimental conditions, αSMA and NG2 expression overlapped in pericytes lining the exterior of vessel walls and in some intra-parenchyma fibrovascular structures. However, FAP and FSP expression did not correspond to these cells and identified unique populations of TAFs often found as isolated cells in infiltrating stroma.

**Figure 1:**
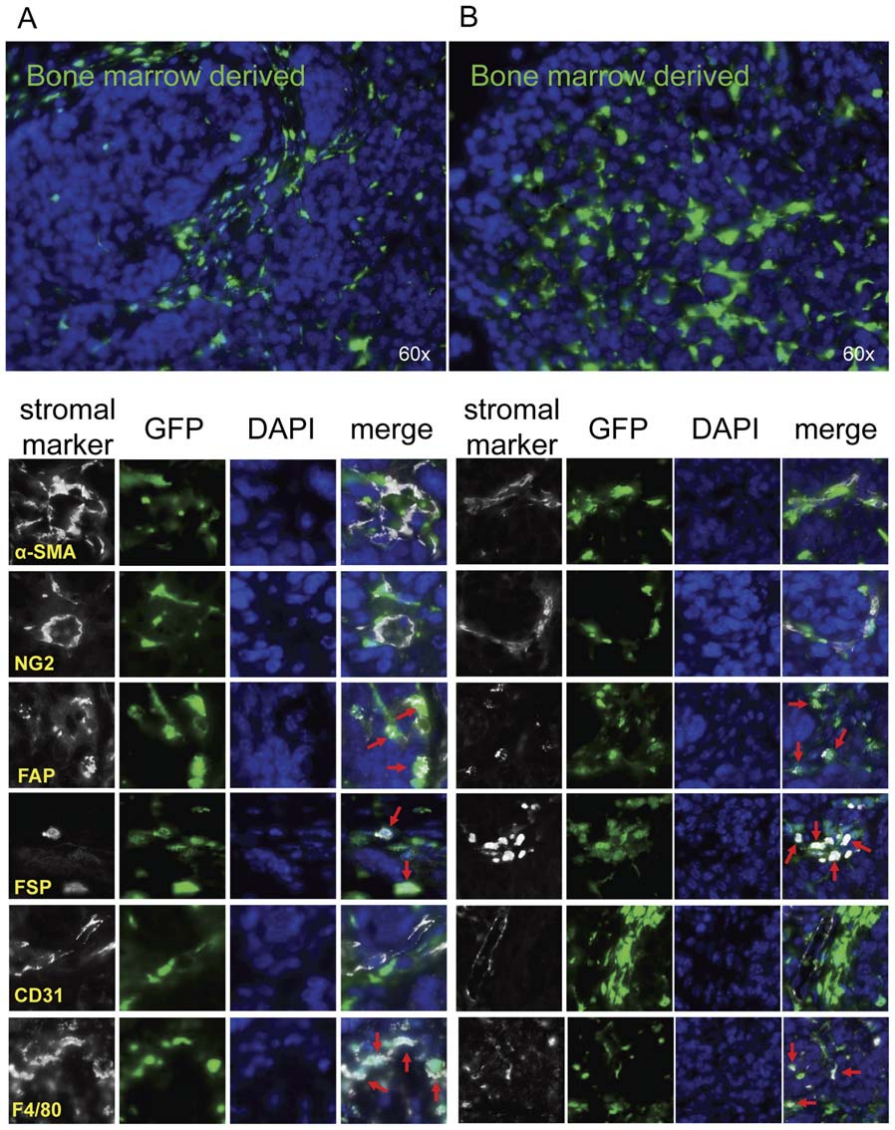
Bone marrow tissue contributions to tumor stromal elements. Lethally irradiated RFP+ mice were reconstituted with GFP+ bone marrow (n = 3). After engraftment, ID8 cells were injected subcutaneously. 5 weeks later, tumors were harvested, and sections were analyzed for α-SMA, NG2, FAP, FSP, CD31, and F4/80 co-staining with GFP+ bone marrow derived cells or RFP+ non bone marrow derived cells. (A&B) Red arrows represent co-staining of GFP+ bone marrow derived cells with FAP, FSP or F4/80. Representative images are shown for 2 separate animals.

**Figure 2:**
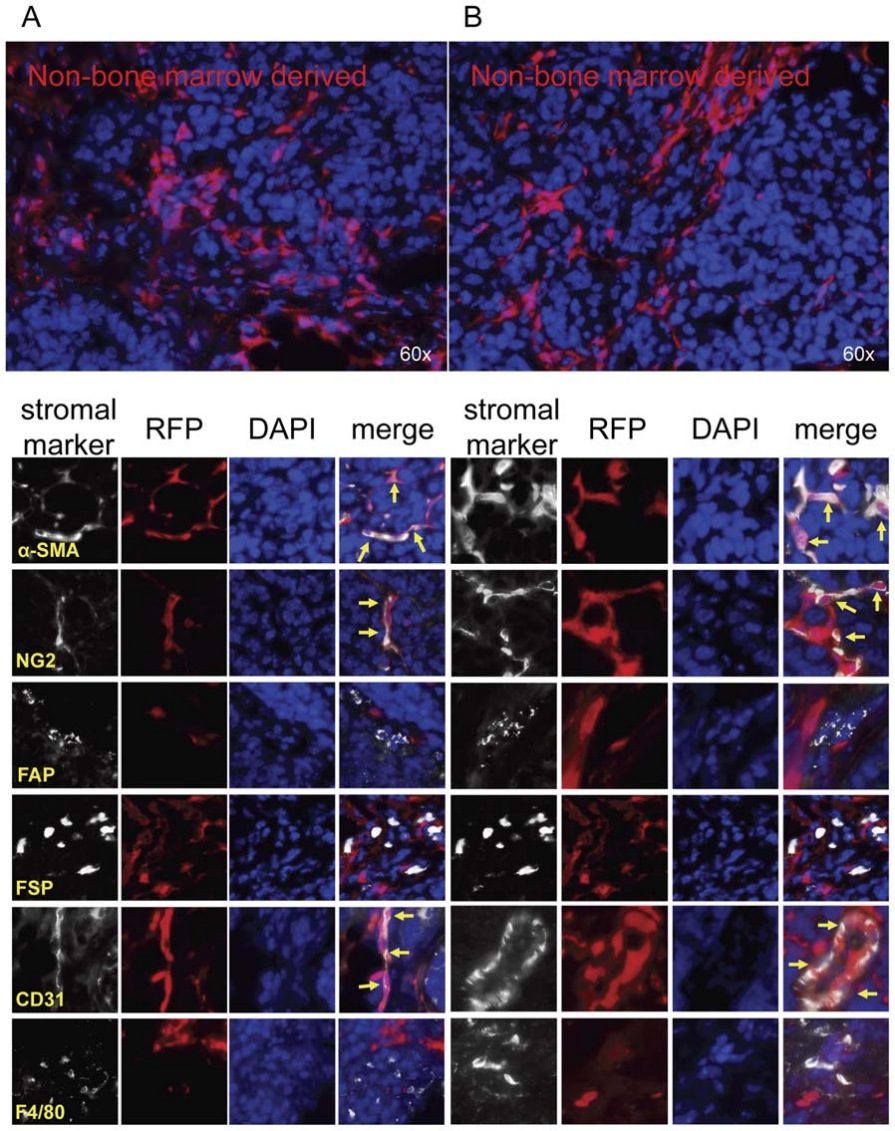
Non-bone marrow tissue contributions to tumor stromal elements. Lethally irradiated RFP+ mice were reconstituted with GFP+ bone marrow (n = 3). After engraftment, ID8 cells were injected subcutaneously. 5 weeks later, tumors were harvested, and sections were analyzed for α-SMA, NG2, FAP, FSP, CD31, and F4/80 co-staining with GFP+ bone marrow derived cells or RFP+ non bone marrow derived cells. (A&B) Yellow arrows represent co-staining of RFP+ non-bone marrow derived cells with α-SMA, NG2 or CD31. Representative images are shown for 2 separate animals.

After quantitation of acquired images stained for bone marrow-derived GFP^+^ cells, host-derived RFP^+^ cells, and markers of stromal cells with Inform software, the origin of each recruited stromal cell population was determined and there were statistically significant (*p*<0.0001) differences between the origin within each stromal marker group (Figure 1 and 2, Table 1). αSMA^+^ cells appeared to be largely non-bone marrow derived in origin (71+/−1%); however, a minor component of bone-marrow derived αSMA^+^ cells were detected representing 20+/−7% the total αSMA^+^ stromal population. Similar to αSMA staining patterns, NG2+ populations were nearly identical in tissue origins: 73+/−5% originating from non-bone marrow tissue and 21+/−8% from bone marrow (Figure S2).

**Table 1:** Quantitative analysis of the origin of stromal elements.

Stromal Marker	Bone marrow derived	Non-bone marrow derived
α-SMA*	20+/−7	71+/−1
NG2*	21+/−8	73+/−5
FAP*	72+/−5	16+/−11
FSP*	63+/−6	23+/−8
CD31*	2+/−1	91+/−6
F4/80*	91+/−6	1+/−1

Table representing the quantitative analysis of the origin of stromal elements. α-SMA+, NG2+, FAP+, FSP+, CD31+, and F4/80+ populations within the tumor microenvironment were analyzed for GFP and RFP co-staining. Numbers represent the percent of the indicated stromal population that were bone marrow derived (GFP+) or non-bone marrow derived (RFP). The percentages were averaged among 3 different animals and are displayed as average +/− standard deviation. P-values (*p<0.0001) were obtained by Student's t-test, N represents total number of nuclei per cluster of images (180,000).

In contrast to αSMA+ and NG2+ pericytic and myofibroblastic populations, fibroblastic FAP^+^ and FSP^+^ cells were more heavily recruited from the bone marrow than from the neighboring adipose tissue (*p*<0.0001). In particular, 72+/−5% FAP^+^ cells were bone marrow in origin while only 16+/−11% originated from non-bone marrow tissues. Correspondingly, 63+/−6% and 23+/−8% of FSP+ cells arose from bone marrow and non-bone marrow tissues, respectively (Table 1).

CD31+ endothelial cells were also quantitated in these analyses. Surprisingly, nearly every vessel (*p*<0.0001) was of non-bone marrow origin (91+/−6%), and only a minority of endothelial cells were possibly bone marrow-derived (2+/−1%). As a positive control, macrophages were also measured and expected to be 100% bone marrow derived if the transplantation were complete. As anticipated, F4/80+ cells were mostly (*p*<0.0001) of bone marrow origin, quantified as 91+/−6% of all macrophages found in the tumor microenvironment. Conversely only 1+/−1% of F4/80+ cells were non-bone marrow derived (Table 1).

### Contribution of endogenous bone marrow derived MSC to the tumor microenvironment

We hypothesized BM MSC may be the cell source of bone marrow derived stromal contribution to the TAF population. Therefore, contribution of BM MSC to the endogenous tumor microenvironment was evaluated by transplantation of both prospectively isolated and *in vitro* isolated and expanded BM MSC prior to tumor development. Lin^−^CD31^−^Sca-1+ cells were prospectively isolated from the bone marrow cells of an RFP expressing mouse. This population was reported by Short *et al.* to contain all MSC activity from the bone marrow [31].

Additionally, mMSC were isolated by *in vitro* plastic adherence. Both populations were verified to express a MSC phenotype and to differentiate into bone, fat and cartilage under appropriate culture conditioning (Figure S3).

**Figure 3:**
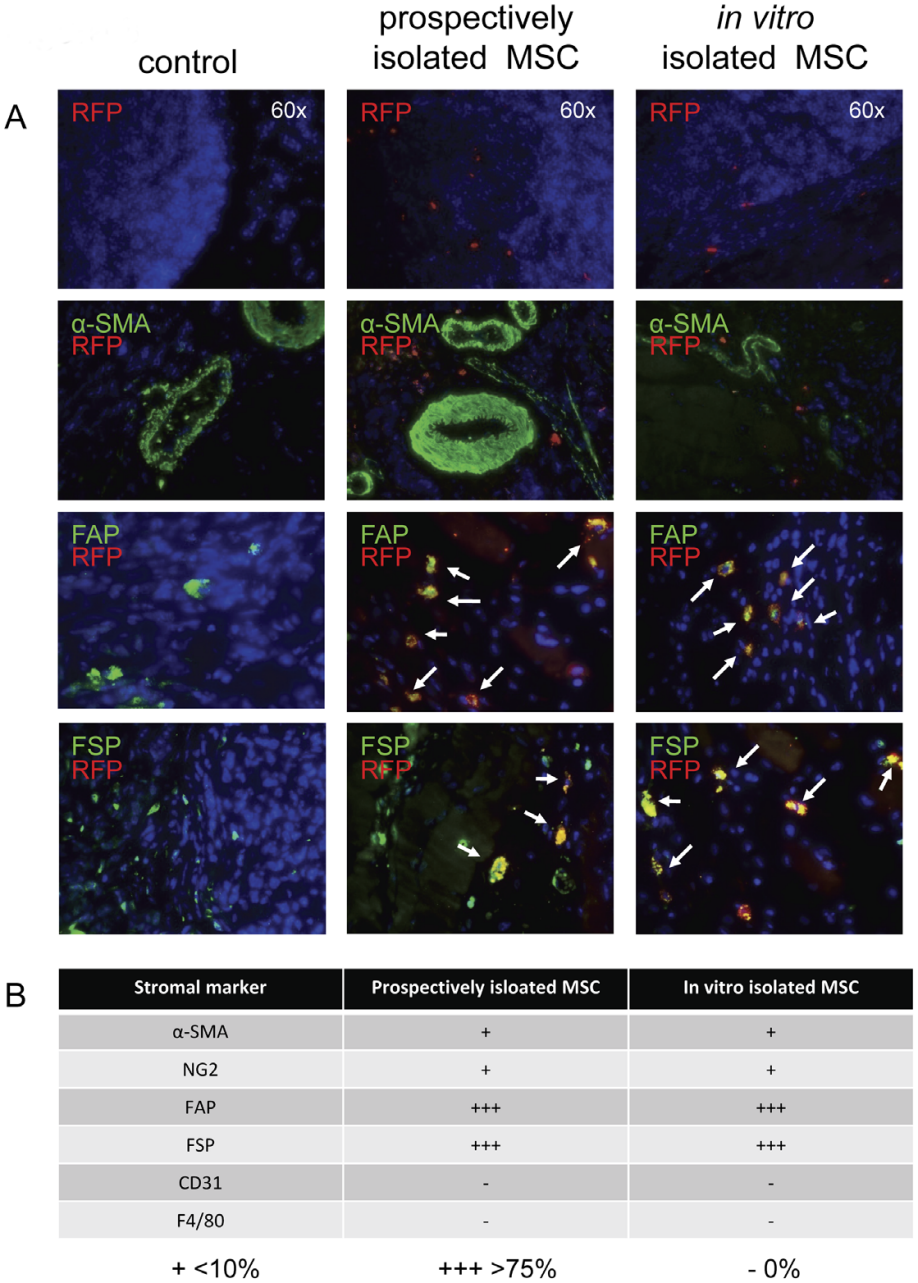
BM MSC contribution to the tumor microenvironment. Prospectively isolated and *in vitro* isolated MSC (RFP+) were combined with whole marrow and transplanted into lethally irradiated mice (n = 3 for each of the 2 groups). After engraftment, ID8 cells were subcutaneously injected. After 5 weeks of tumor groups, sections from harvested tumors were analyzed for RFP+ cells. (A) Representative images are shown for RFP co-staining with FAP and FSP as indicated by the white arrows in the merged images. There is no co-staining between RFP+ cells and α-SMA. (B) Scores were assessed for the relative percentage of RFP+ cells co-staining with each stromal marker.

RFP+ cells were detected in the tumor microenvironments of both prospectively isolated and *in vitro* cultured BM MSC transplant recipients (Figure 3). In all samples, recruited RFP+ cells were most frequently identified around the tumor periphery as isolated cells with no apparent vascular or fibrovascular structural formation. The spatial organization was similar to that of FAP+ and FSP+ detected cells in the whole bone marrow transplant experiments sections. Correspondingly, >75% of both prospective and *in vitro* isolated BM MSC populations (the sorted RFP+ cells and *in vitro* RFP+ MSC) that were recruited to the tumor microenvironment co-expressed FAP and FSP (Figure 3). In accord with the apparent lack of RFP+ fibrovascular structures, most RFP+ populations were negative for α-SMA and NG2, though a few positive cells could be detected in each sample. None of the RFP+ transplanted populations co-stained with CD31, indicating they did not contribute to the endothelial compartment of the tumor microenvironment.

### Contributions of adipose-derived stromal cells in E0771 breast tumors

We next hypothesized that cells in neighboring adipose tissue may account for non bone marrow tissue contribution to TAFs (pericyte and myofibroblasts) and/or endothelial populations. Furthermore, we sought to verify this non bone marrow tissue contribution in another tumor model and examined E0771 breast tumors. Therefore, mice were subcutaneously transplanted with GFP+ adipose tissue, and after 10 days to facilitate engraftment, E0771 murine breast cancer cells were injected adjacent to transplanted adipose tissue. Upon termination of the experiment, tumor and surrounding adipose tissues were removed and analyzed.

**Figure 4:**
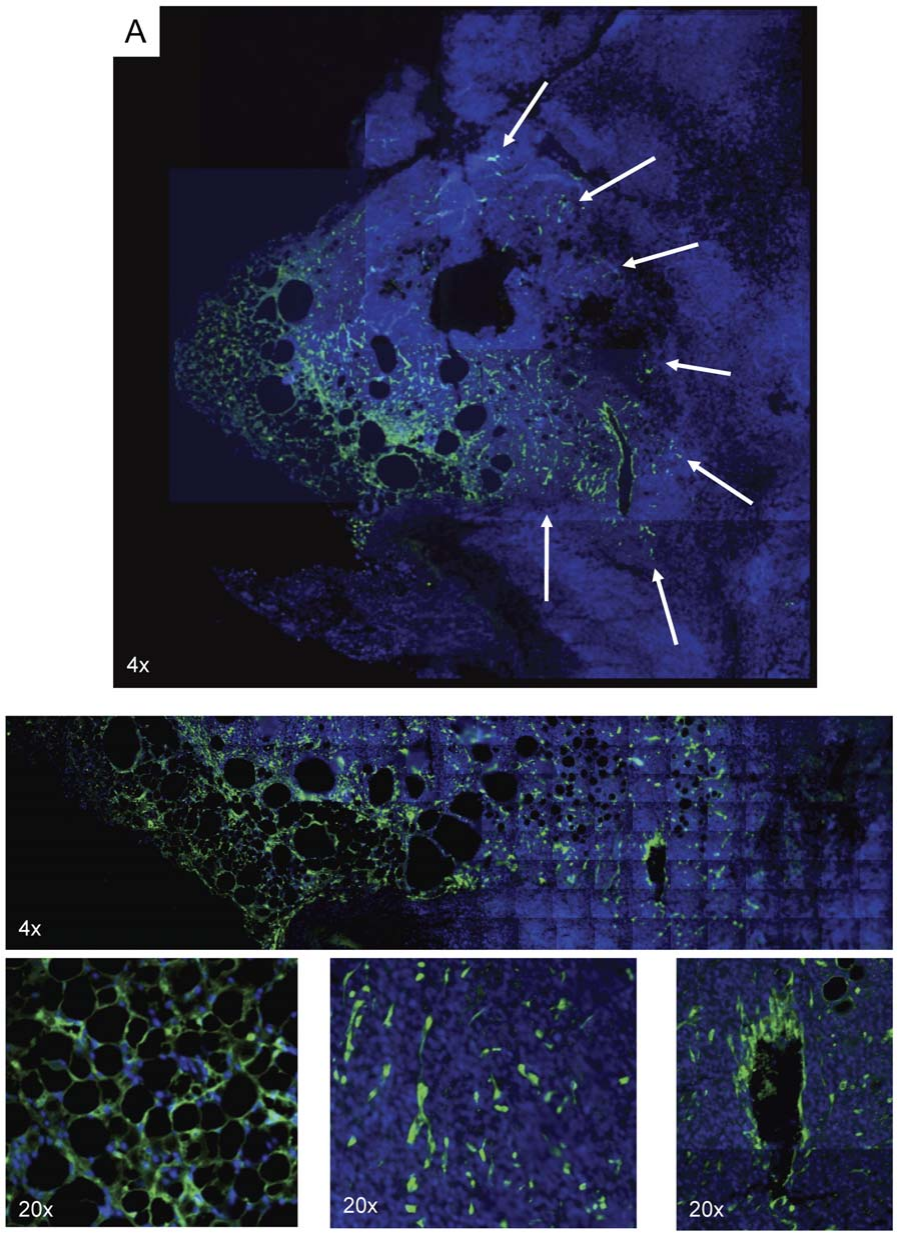
Engrafted GFP+ adipose tissue is locally recruited into the tumor microenvironment. GFP+ adipose tissue was subcutaneously implanted into wild type mice (n = 3). After engraftment, E0771 cells were subcutaneously injected. Two weeks later, sections of resected tumors and adjacent adipose tissue were analyzed for GFP expression. Analysis revealed engrafted adipose tissue with normal morphology. Recruited GFP+ adipose derived cells remained in close proximity to the transplanted adipose tissue. White arrows in the montage image indicate adipose-derived tumor stroma participants.

GFP staining of the transplanted adipose tissue indicated the presence of successfully engrafted vascularized adipose tissue bearing typical adipocyte morphology (Figure 4). GFP+ cells were found throughout adjacent, infiltrating tumor tissue but remained proximal to the site of transplanted adipose tissue (Figure S4). Tumor tissue distal to GFP+ adipose tissue displayed no evidence of recruited GFP+ stromal cells.

**Figure 5:**
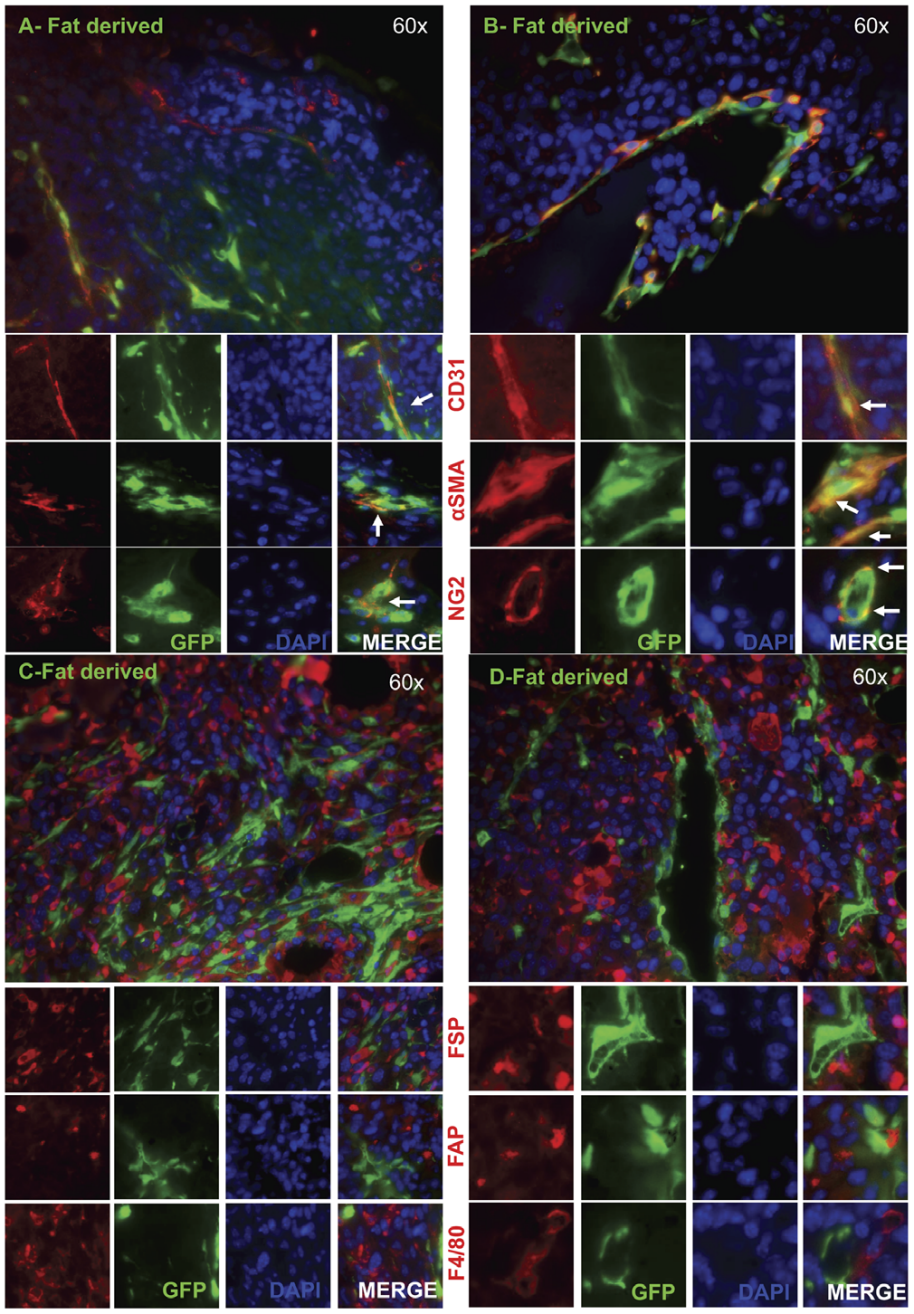
Stromal marker expression of recruited GFP+ adipose derived cells. GFP+ adipose-derived cells displayed (A & B) a high degree of overlap in expression of CD31, α-SMA, and NG2 as indicated by the white arrows in the merged column and (C & D) minimal overlap with FAP, FSP, and F4/80. Representative images are shown for 2 separate animals.

When quantified, it was determined that the transplanted adipose tissue contributed mainly to α-SMA, NG2, and CD31 endothelial cell populations (Figure 5 A&B). Specifically, of the recruited GFP+ cells, 18+/−2% gave rise to CD31+ endothelial cells. 55+/−3% and 58+/−5% of GFP+ cells within the tumor tissue co-stained with α-SMA and NG2, respectively (Table 2). Conversely, only 7+/−3% of the adipose-derived cells were also positive for FSP, and an even smaller percent co-localized with FAP expression (2+/−1%; Figure 5 C&D; Table 2). As a negative control, staining for macrophage marker F4/80 revealed 2+/−1% co-localization with GFP expressing cells.

**Table 2:** Recruited adipose derived cell expression of stromal markers.

Stromal Marker	% fat derived cells co-stained
α-SMA*	56+/−5
NG2*	58+/−8
FAP*	2+/−1
FSP*	7+/−5
CD31*	18+/−3
F4/80*	2+/−1

GFP+ cells recruited from local adipose tissue were quantified within the tumor stroma for co-expression of stromal markers. Numbers presented represent the percentage of the recruited GFP+ cells that were also positive for the indicated marker. The percentages were averaged among 3 different animals and are displayed as average +/− standard deviation. P-values were obtained by Student's t-test, N represents total number of nuclei per group (180,000).

## Discussion

In this work, we quantified the α-SMA+, NG2+, FAP+, FSP+, CD31+, and F4/80+ stromal contributions to the tumor microenvironment. Many previous reports have investigated various bone marrow derived cell recruitment to tumors, however, these studies have focused on a single marker or a single stromal element and few attempted quantitation of these contributions [1], [6], [9], [20], [24], [32], [33]. An elegant series of experiments by Quante *et al.* revealed the incorporation of bone marrow derived αSMA+ cells amounting to about 20% of the tumor stroma [9], similar to our own results where we observed a far greater contribution of αSMA+ tumor stroma from the local adipose tissue than from the bone marrow derived source.

When sub-divided, quantitative results of stromal marker expression indicated that the majority of FSP+, FAP+ and F4/80+ stromal populations found within the tumor microenvironment originated from the bone marrow (*p*<0.01). Conversely, most α-SMA, NG2, and CD31 expressing cells were of a non-bone marrow, adipose tissue-derived origin (*p*<0.01). While α-SMA, NG2, FAP, and FSP have often been grouped together as collective markers for TAF and/or myofibroblasts [7], [27], [29], in this work it is apparent these markers designate disparate mesenchyme populations with distinct tissues of origin. These findings are in accord with reports from Sugimoto *et al.* indicating heterogeneity within the fibroblast compartment of the tumor microenvironment [34]. In examining syngeneic pancreatic and breast tumors, Sugimoto reported significant overlap between α-SMA and NG2 antigens and minimal overlap between α-SMA/NG2 and FSP, concluding that FSP identifies a unique population of fibroblasts within the tumor stromal component. Corresponding distinctions between FSP and α-SMA expression have also been noted in examinations of fibrotic glomerulonephritis [35].

The bone marrow-derived tumor associated stromal components defined by FAP and FSP expression could be largely characterized as isolated cells, lacking organization near the periphery of the tumor. The primary functions of calcium binding protein FSP and dipeptidyl peptidase FAP revolve around promoting migration, altering adhesive properties and remodeling the extracellular matrix [36]–[38]. These properties are most frequently utilized at sites of expansion and metastasis along the periphery of tumor development, which is consistent with our observations.

Conversely, non-bone marrow derived cells were often found in clusters, demonstrating organization into vascular and fibrovascular-like structures. In our model, α-SMA and NG2 expression localized to cells organized into vessel-like formations, corresponding to pericytic locations throughout the tumor. These spatial distribution findings are consistent with a report from Udagawa et al. indicating a similar pattern in recruited bone marrow cells to Lewis lung carcinoma tumors [1]. In our work herein, fairly equal distribution (p>0.05) of bone-marrow versus non-bone marrow derived stroma was observed as a total when looking at GFP+ versus RFP+ stroma within the tumor microenvironment.

One very striking result of the transplant experiments was a notable lack of bone marrow originating CD31+ endothelial cells. The existence of a circulating bone marrow-derived endothelial progenitor (BM EPC) has been a topic of controversy for the past decade, dating back to its initial description by Asahara and colleagues in 1997 [39]. BM EPC have been reported to contribute significantly to tumor vasculature in many investigations [6], [32], [33]. However, other studies have revealed no contribution of the bone marrow to neovasculature in tumors [18], [24], [40], [41]. It is likely that the array of conflicting results has arisen from a multitude of experimental conditions differing in each investigation. In fact, work by Monsky et al. illustrated this point. In their study, the degree of bone marrow derived endothelial progenitor cell incorporation in mammary tumor vasculature varied from <4% when implanted in the fat pad or subcutaneous space to nearly 60% when implanted in the brain [42]. Their results also varied greatly among different tumor types and mouse strains. Our results are in accord with experiments in this study utilizing subcutaneously implanted syngeneic C57Bl/6 lung carcinoma and melanoma tumors where minimal bone marrow derived endothelial cell contributions were noted. However, under alternative conditions such as orthotopic implantation or a different time course, the outcome may have been different.

In the next set of experiments, we examined the potential of prospectively isolated BM-MSC to give rise to the bone marrow derived stromal components in the tumor microenvironment. Though publications utilizing prospectively isolated murine MSC are rare, a recent investigation has identified prospective these cells within the Lin^−^CD31^−^Sca-1^+^ fraction of bone marrow mononuclear cells [31]. Similar experiments have been done based on αSMA+ fractionation of MSC to reveal the contribution of αSMA-expressing MSC to the tumor stroma in gastric cancer [9].

We found transplanted cells from this population as well as BM MSC isolated by traditional methods of plastic adherence and *in vitro* expansion were both recruited to the tumor microenvironment. Flow cytometric analysis of peripheral blood and whole bone marrow from transplanted animals showed full donor chimerism (<1% host-derived cells in the peripheral blood). Immunofluorescence of adipose tissue sections, however, did show the presence of prospective and *in vitro* expanded BM MSC. Since MSC are resistant to radiation, as evidenced by their persistence after a lethal dose in our hands and previous reports [43], [44], these cells are still occupying the bone marrow MSC niche, and transplanted MSC may not have a place to engraft within that environment. Adipose tissue is known to contain what is termed as adipose derived stem cells (ASC) or adipose derived MSC, which appear similar to BM MSC in many regards [45]–[48]. Additionally, this tissue readily expands, perhaps providing new potential MSC niches [49].

Both prospective and *in vitro* isolated MSC populations were found at similar rates of incidence within the tumor microenvironment, typically localized to the tumor mass periphery. 64–76% of recruited BM MSC were identified in stromal locations, and only 23–35% were found within the tumor parenchyma. Correspondingly, co-staining with stromal markers revealed similar phenotypes within the tumor microenvironment. Most of these cells were also positive for FSP and FAP. A minority of recruited cells in each group was found to express α-SMA and NG2, and all RFP+ recruited cells were negative for CD31 and F4/80. These results indicate that *in vitro* expanded BM MSC and Lin^−^CD31^−^Sca-1^+^ prospective BM MSC are a potential source of bone marrow derived FAP+ and FSP+ stromal cells recruited to the tumor microenvironment.

In search of the source of tumor associated myofibroblasts, pericytes and endothelial cells not recruited from the bone marrow, we next hypothesized these elements may be arising from neighboring adipose tissue. Obesity has been determined as a predisposing factor for cancer development and is associated with poor prognosis of certain cancer types [50]. Since obesity arises from overgrowth of white adipose tissue (WAT), it has been proposed that this tissue may play a significant role in tumor initiation and/or progression [4], [51], [52]. WAT is composed of many cell types, including adipocytes, pre-adipocytes, endothelial cells, pericytes and an assortment of stromal cells [49]. Precursors from WAT have been shown to contribute to *in vitro* vessel formation and stabilization [48] as well as *in vivo* revascularization of damaged skeletal muscle [53]. Within the stromal fraction of WAT, progenitors with a similar phenotype, proliferative rate and differential capacity to BM MSC have been identified [47], [54]. Recently, exogenous addition of these progenitors, referred to as adipocyte stem or stromal cells (ASC), has been shown to enhance tumor progression in syngeneic and xenogeneic models [4], [8]. Additionally, adipose tissue transplanted in a nude mouse was shown to be recruited to developing tumors 2cm away from the fat location [4], [8]. Taken together, these findings suggest a role for adipose-derived cells in the tumor microenvironment, though their participation has not been fully investigated.

In our studies, GFP+ adipose tissue was successfully engrafted as indicated by vascularization, typical adipocyte morphology and size to control adipose tissue specimens, as previously described [55]. Interestingly, GFP+ adipose-derived cells visualized within the tumor bulk were not uniformly distributed; only tumor tissue within close proximity to the transplanted adipose tissue (<5 mm) contained GFP+ cells. Parts of the tumor distal (>5 mm) to the site of GFP+ adipose tissue transplantation were negative for GFP+ recruited stromal components. These findings are in accord with adipose tissue transplantation results reported by Zhang *et al.*, though in their nude mouse model with low levels of endogenous adipose tissue, transplanted adipose derived cells traveled much greater distances (>2 cm). C57Bl/6 mice utilized in our model did contain endogenous adipose tissue, and it is likely recruitment of endogenous adipose derived cells could account for many stromal components distal to the site of GFP+ adipose tissue transplantation. Both of these models support the hypothesis of a role for adipose-derived cells in the tumor microenvironment. However, the difference seen between them suggests that tumor behavior is largely dictated by environmental context. Models with low levels of endogenous adipose tissue may not accurately reflect the disease seen in the average human, since nearly 70% of adults in the U.S. are classified as overweight or obese [56].

Quantitation of GFP+ recruited adipose derived cells revealed that most (50–60%) of these cells were positive for α-SMA and NG2. The location and structural formation of these double positive cells also corresponded to perivascular areas. Additionally, nearly 20% GFP+ adipose derived cells were positive for CD31 and formed vessel structures within the tumor parenchyma. Varying reports in the literature have indicated conflicting data concerning the origins of tumor associated stromal cells [1], [7], [15], [20], [25], [57], [58]. It is likely these discrepancies stem from variations in experimental models and design. As mentioned previously, Monsky and colleagues described a <4% incorporation of bone marrow derived endothelial progenitors when a breast tumor was implanted in or near adipose tissue, yet this number rose to 60% when the same tumor was located in the brain [42].

**Figure 6:**
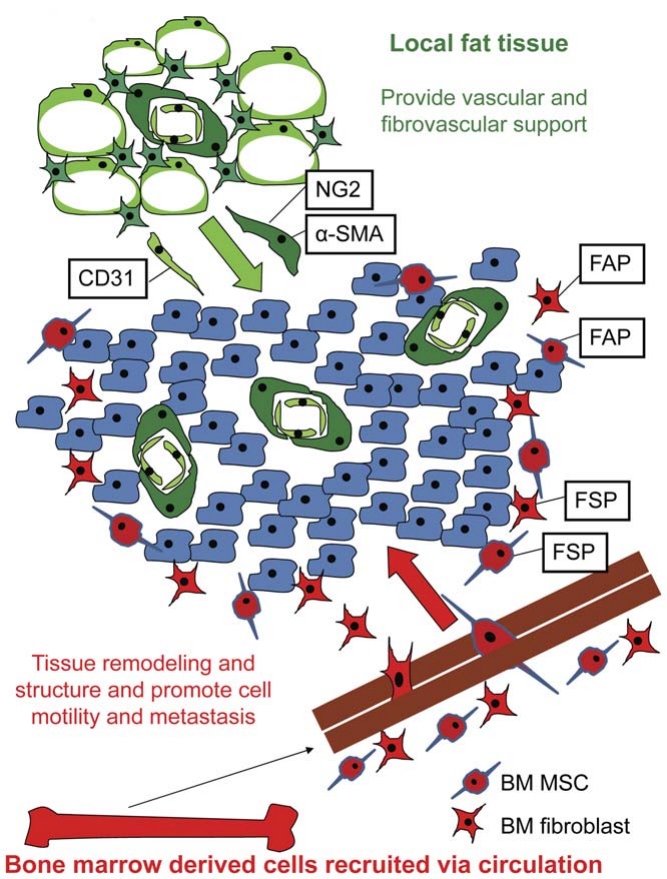
Model of stromal recruitment. A tumor is composed of not only cancer cells, but also recruited host-derived cells. Our model suggests that the majority of pericytes (NG2+ and α-SMA+) and endothelial cells (CD31+) are recruited from local tissue, such as local adipose tissue. FAP+ and FSP+ fibroblastic cells involved in extracellular matrix remodeling are recruited from host bone marrow populations, such as BM MSC.

These results along with our own would suggest that tumors will preferentially recruit stromal cells from nearby tissue such as fat when it is available. However, when unavailable, tumors may resort to recruitment from alternate and often more distant sources. The term “tumor associated fibroblast” has been used to describe cell populations identified by a variety of phenotypic markers [7], [27]–[29]. Though TAFs have been collectively grouped under the same generic nomenclature, our results as well as the results of others [34] indicate this is a heterogeneous population with discreet subpopulations. Our results further indicate different TAFs have distinct tissues of origin. In our model, we propose that when available, tumors will recruit vascular endothelial cells and fibrovascular TAFs or pericytes as defined by αSMA and NG2 from nearby, local, adipose tissue (Figure 6). However these locally recruited cells cannot meet the tumor's tissue remodeling needs as it expands and grows. At this point, another subpopulation of TAFs is recruited systemically from the bone marrow. Our data confirm that BM MSC are a source of bone-marrow derived cells and once they are in the tumor microenvironment, a subset will express pathological disease associated markers FSP and FAP. These proteins mark the transformation of a recruited fibroblastic cell into a pathological cell which aids in the promotion of tumor extracellular matrix remodeling, motility, and metastasis.

Our model suggests that the basis for tumor stroma heterogeneity arises from the disparate origin of the stromal components, however differing tumor types, modes of tumor establishment, age of the host and time of evaluation are likely to alter the microenvironment composition. For example, bone marrow derived cells may contribute more heavily to pericyte and/or endothelial populations when local cells cannot meet the growth demand of the tumor vasculature. As we have learned in recent years, the tumor is composed not only of transformed cancerous cells, but also normal cells recruited to aid in tumor growth and propagation. The role of these key players and their interactions in the tumor microenvironment reveals a new paradigm for cancer treatment by targeting the “soil” instead of only the “seed.” The identification of this cellular milieu is the first step in unraveling the complex interactions between tumor and host cells and identifying possible areas for intervention. The models we have developed have not only revealed insights to the composition of the tumor microenvironment but also provide a platform on which candidate drugs aimed at disrupting tumor-stroma interactions may be tested.

## Materials and Methods

### Cell Culture

ID8 C57Bl/6 murine ovarian tumor cells were a generous gift of Dr. Kathy Roby (University of Kansas Medicial Center) [59]. The cells were maintained in DMEM supplemented with 10% FBS, penicillin-streptomycin, and L-glutamine. The E0771 C57Bl/6 murine mammary tumor cells (a kind gift from Dr. F.M. Sirotnak, Memorial Sloan Kettering, New York, NY) were maintained in RPMI 1640 supplemented with 10% FBS, 10 mmol/L HEPES, penicillin-streptomycin, and L-glutamine.

### *In vitro* MSC Isolation

Murine MSC (mMSC) were isolated as described previously [14]. Briefly, femurs of 2-month-old C57Bl/6 mice (Harlan Labs, ME) were collected, dissected into small fragment, then placed into a sterile mortar and crushed using a sterile pestle. Bone marrow was reserved, and bone fragments were incubated with Type I collagenase at 37°C. After incubation, liberated cells were combined with reserved marrow and plated in αMEM with 20% FBS in a 180 cm^2^ dish. After five days, the plate was washed to remove non-adherent cells. Adherent cells were retrieved by trypsinization and immunodepleted of granulo-monocytic cells using a biotinylated antibody against CD11b (BD Biosciences, San Jose, CA), and streptavidin-coated microbeads from Miltenyi Biotec (Auburn, CA) according to the manufacturer's instructions. After immunodepletion, the remaining cells were plated in fresh media, and within 3 additional days, fibroblast-like colonies were observed. Medium was changed two to three times a week and cell density was maintained between 2,000 and 6,000cell/cm^2^.

### Prospective MSC Isolation

Prospective MSC were isolated as previously described with some modification [31]. Whole bone marrow was collected from C57Bl/6 mice and enriched for Sca-1+ cells with MACS microbeads, as recommended by the manufacturer (Miltenyi Biotec, Auburn, CA). Post enrichment, cells were stained with APC conjugated rat anti mouse hematopoietic lineage antibodies (CD3, CD4, CD5, CD8, CD11b, Gr-1, B220, and Ter-119) and APC conjugated rat anti-mouse CD31 (BD Biosciences, San Jose, CA). Fluorescence activated cell sorting was then performed to isolate Lin−CD31−Sca-1+ cells on a BDFACS Aria (Becton Dickenson, San Jose, CA).

### Animals

Transgenic C57Bl/6 mice expressing either GFP under the control of the ubiquitin promoter, RFP under the control of the chicken beta actin promoter or LacZ under the control of the ROSA26 promoter were purchased from Jackson Labs, and bred in house to maintain colonies. Mice were utilized for experiments between 8 and 12 weeks of age. All mice were housed and treated in accordance with institutional standards. This study was approved by the MD Anderson Institutional Review Board and Institutional Animal Care and Use Committee approved protocol (100510632).

### Whole Bone Marrow Transplantation

Whole marrow was isolated from the tibia, femur, and iliac crest, as detailed in the “Isolation and propagation of murine MSC” section above. Recipient C57Bl/6 mice were lethally irradiated with 10 Gy 4 hours before reconstitution. 10^7^ donor whole marrow cells suspended in 100 µl PBS were then tail vein injected (IV) into irradiated mice. Control animals received PBS injections. Within 2–3 weeks, control animals died while transplanted animals survived and displayed 99% donor-derived cells in the bone marrow.

### MSC Transplantation

MSC were isolated prospectively and *in vitro* as described above from an RFP+ mouse. 100,000 of each population was combined with 10^7^ whole marrow cells from a LacZ+ mouse and transplanted into a lethally irradiated GFP+ recipient as described in whole marrow transplantation.

### Adipose tissue Transplantation

Adipose tissue transplants were performed as described previously [55]. Donor fat pads were removed from the intra-abdominal perigonadal area of GFP+ mice. The fat pads were sliced into 100–150 mg pieces and stored in warm PBS until the time of transplant. The right dorsal side of anesthetized mice was shaved and cleaned with alcohol. Fat slices were placed just below the skin in the subcutaneous space, and incisions were closed with metal clips. Clips were removed when the wound was resolved 3–5 days after transplant.

### Tumor Administration

After transplantation procedures were performed and tissue engraftment was verified, tumor cells were subcutaneously injected into the upper hind limbs in the case of bone marrow transplantation experiments. Tumor cells were subcutaneously injected adjacent to the site of adipose tissue transplantation on the backs of recipients. ID8 tumors were established and harvested 5 weeks after injection of 10^7^ ID8 tumor cells. E0771 tumors were established and harvested 2 weeks post injection of 5×10^4^ E0771 tumor cells.

### Flow Cytometry

Cells were resuspended in PBS supplemented with 2% FBS (10^6^ cells/100 µl/staining reaction). 1 µg of each antibody was added to the cell suspension and incubated at 4°C for 30 minutes. Labeled cell populations were then analyzed on an LSR II flow cytometer (Becton Dickenson, San Jose, CA) with FACS Diva software. Sample acquisition was accompanied with use of control unstained, single color stained and isotype controls to determine the appropriate voltages, compensations, and positioning of gates for data acquisition.

### Immunofluorescence

Paraffin embedded sections were rehydrated and deparaffinized. Primary antibodies used for fibroblast detection were rabbit anti-fibroblast activation protein (abcam) and rabbit anti-S100A4/fibroblast specific protein (Dako). Myofibroblasts were identified by mouse IgG2a anti-α-smooth muscle actin (abcam) and rabbit anti-NG2 (Chemicon). Antibodies for endothelial and macrophage detection were rabbit anti-CD31 (abcam) and F4/80 (abcam), respectively. Secondary antibodies conjugated to AlexaFluor350, AlexaFluor488, AlexaFluor594, and AlexaFluor647 fluorochromes (Invitrogen) were used for primary antibody detection. Nuclei were identified by DAPI staining.

### Image Acquisition and Data Analysis

Stained slides were mounted with Dako Anti-fade mounting medium (Dako) and visualized on an Olympus IX51. Multi-spectral data was acquired with Nuance camera and imaging software. Data analysis was performed with Inform software (Figure 3). First, regions of interest were defined on 4–6 images, and the recognition software was trained to classify all images. Then, nuclei were located based on DAPI fluorescence and defined nuclear size parameters within the classified area of interest. Next, cytoplasm was drawn around the identified nuclei as described by user-defined parameters, and then fluorescence data in pixels was quantified for each nucleus and cytoplasm for each cell. Data was exported into excel where nuclear and cytoplasmic signals were summed to give per cell quantitation of pixel count for each fluorochrome. Numerical cutoffs based on isotype controls were used to define Alexa fluor 488+, Alexa fluor 594+, and double positive cell populations, and each image was evaluated on a percent positive basis (Figure 4). 10 images per slide were quantitated and averaged at 3 different depths within the tumor, which were in turn averaged to give a final percent across each tumor. 3 tumors were analyzed in this manner per experimental group.

### Statistical Analysis

Results are reported as means +/− standard error. P-values were obtained by Student's t-test. N was number of nuclei per group based on dapi staining and was on average 60,000 per animal and 180,000 stromal marker group based on 3 replicates per group.

## Supporting Information

Figure S1 **Bone marrow transplant experimental design.** Bone marrow from a GFP+ mouse was transplanted into a lethally irradiated RFP+ mouse. After 4 weeks, engraftment is verified by >99% GFP positivity in peripheral blood as well as bone marrow. At this time, ID8 cells are injected subcutaneously. After 5 weeks of tumor development, the tumor is resected and analyzed for recruited bone marrow (GFP+) and non bone marrow (RFP+) host derived cells in the tumor microenvironment.
(TIF)

Figure S2 **RFP+ Bone marrow and GFP+ non-bone marrow tissue contributions to the tumor microenvironment.** To verify results, the converse bone marrow transplantation experiment was performed in which lethally irradiated GFP+ mice were reconstituted with RFP+ bone marrow (n = 3). After engraftment, ID8 cells were injected subcutaneously. 5 weeks later, tumors were harvested, and sections were analyzed for α-SMA, NG2, FAP, FSP, CD31, and F4/80 co-staining with (A) RFP+ bone marrow derived cells co-stain with FAP, FSP and F4/80 as depicted by the yellow arrows in the merge column. (B) GFP+ non bone marrow derived cells co-stain with α-SMA, NG-2 and CD31 as identified by the red arrows in the merge column. Representative images are shown from 1 animal.
(TIF)

Figure S3 **Characterization of MSC.** mMSC were isolated by (A) *in vitro* plastic adherence or (B) prospective sorting of Lin− CD31− Sca-1+ cells. Cells from both populations were placed in culture and analyzed for bone, fat, and cartilage differentiation potentials as evidenced by Alizarin Red S, Oil Red O, and Alcian Blue staining, respectively. They were also phenotypically examined for CD44, Sca-1, CD140b, and CD106 expression and a lack of CD45, CD11b, and CD31.
(TIF)

Figure S4 **Engrafted GFP+ fat is locally recruited into the tumor microenvironment.** GFP+ fat was subcutaneously implanted into wild type mice (n = 3). After engraftment, E0771 cells were subcutaneously injected. Two weeks later, sections of resected tumors and adjacent fat were analyzed for GFP expression. Analysis revealed engrafted fat with normal morphology. Recruited GFP+ fat derived cells remained in close proximity to the transplanted fat.
(TIF)
