# Supplementary figures and images for: Origins of the Tumor Microenvironment: Quantitative Assessment of Adipose-Derived and Bone Marrow–Derived Stroma
Source: PLoS One. 2012 Feb 20;7(2):e30563. doi: 10.1371/journal.pone.0030563 (PMC3282707; doi:10.1371/journal.pone.0030563)

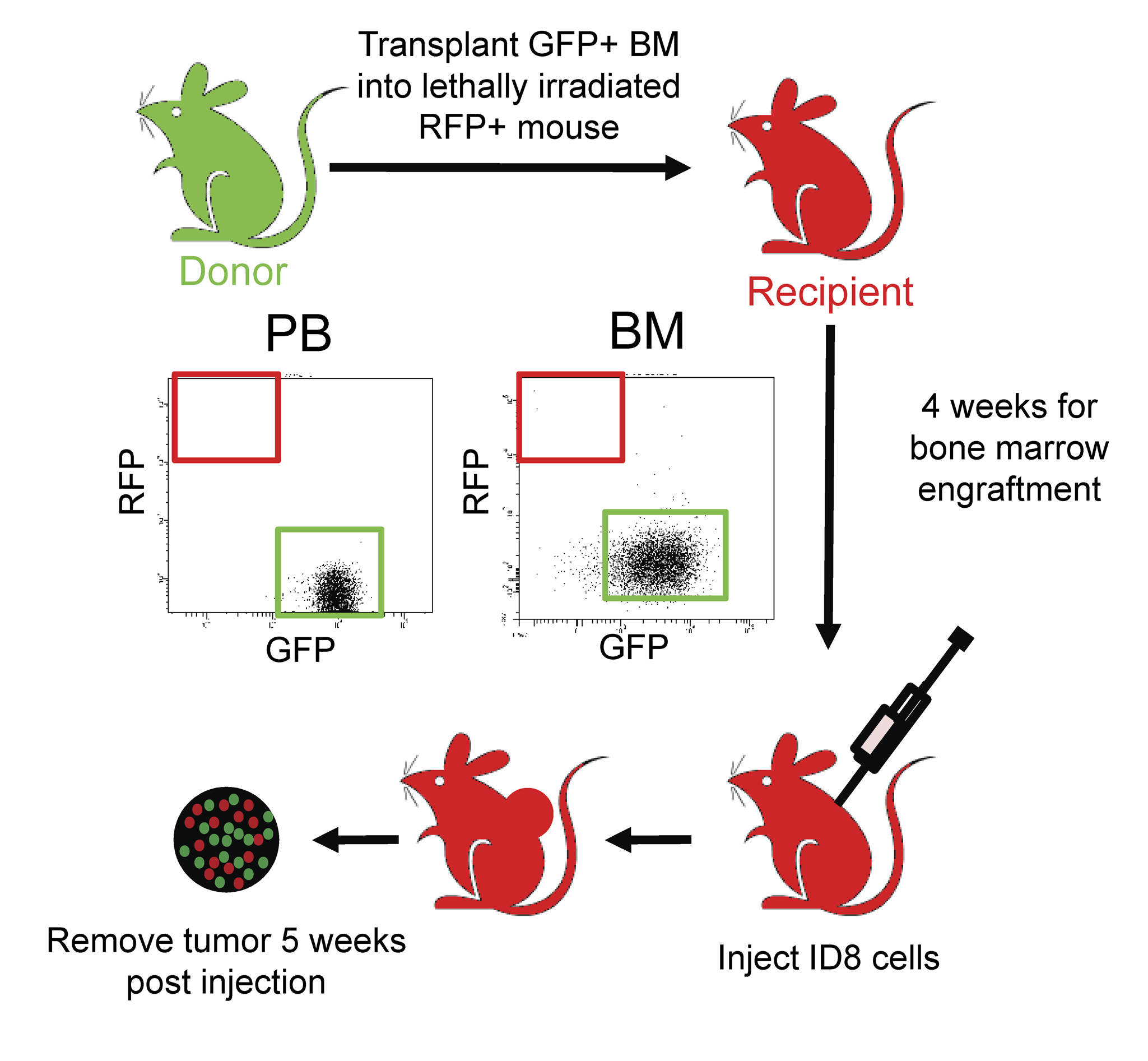

Supplement: Figure S1 — Bone marrow transplant experimental design. Bone marrow from a GFP+ mouse was transplanted into a lethally irradiated RFP+ mouse. After 4 weeks, engraftment is verified by >99% GFP positivity in peripheral blood as well as bone marrow. At this time, ID8 cells are injected subcutaneously. After 5 weeks of tumor development, the tumor is resected and analyzed for recruited bone marrow (GFP+) and non bone marrow (RFP+) host derived cells in the tumor microenvironment. (TIF) [file pone.0030563.s001.tif]

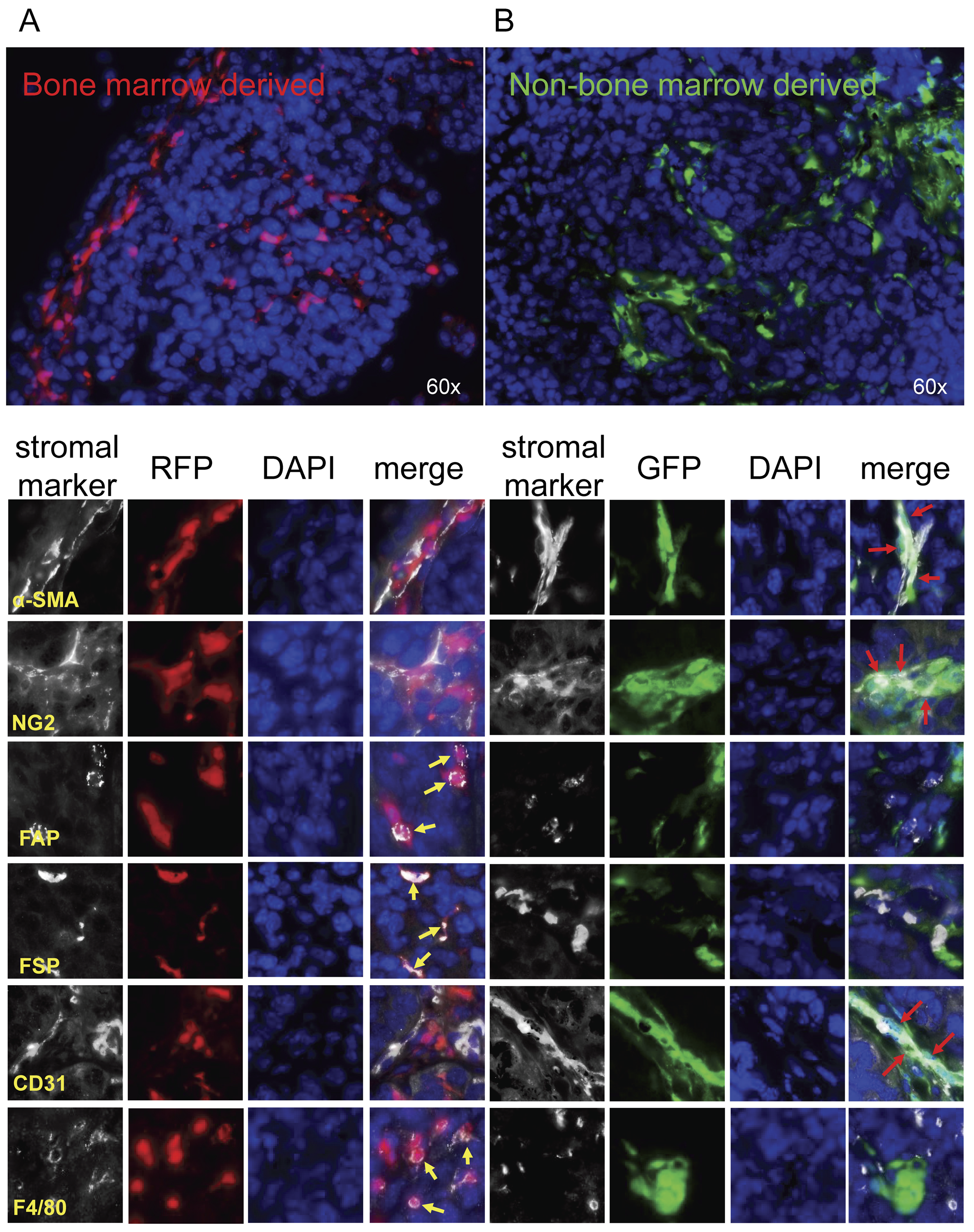

Supplement: Figure S2 — RFP+ Bone marrow and GFP+ non-bone marrow tissue contributions to the tumor microenvironment. To verify results, the converse bone marrow transplantation experiment was performed in which lethally irradiated GFP+ mice were reconstituted with RFP+ bone marrow (n = 3). After engraftment, ID8 cells were injected subcutaneously. 5 weeks later, tumors were harvested, and sections were analyzed for α-SMA, NG2, FAP, FSP, CD31, and F4/80 co-staining with (A) RFP+ bone marrow derived cells co-stain with FAP, FSP and F4/80 as depicted by the yellow arrows in the merge column. (B) GFP+ non bone marrow derived cells co-stain with α-SMA, NG-2 and CD31 as identified by the red arrows in the merge column. Representative images are shown from 1 animal. (TIF) [file pone.0030563.s002.tif]

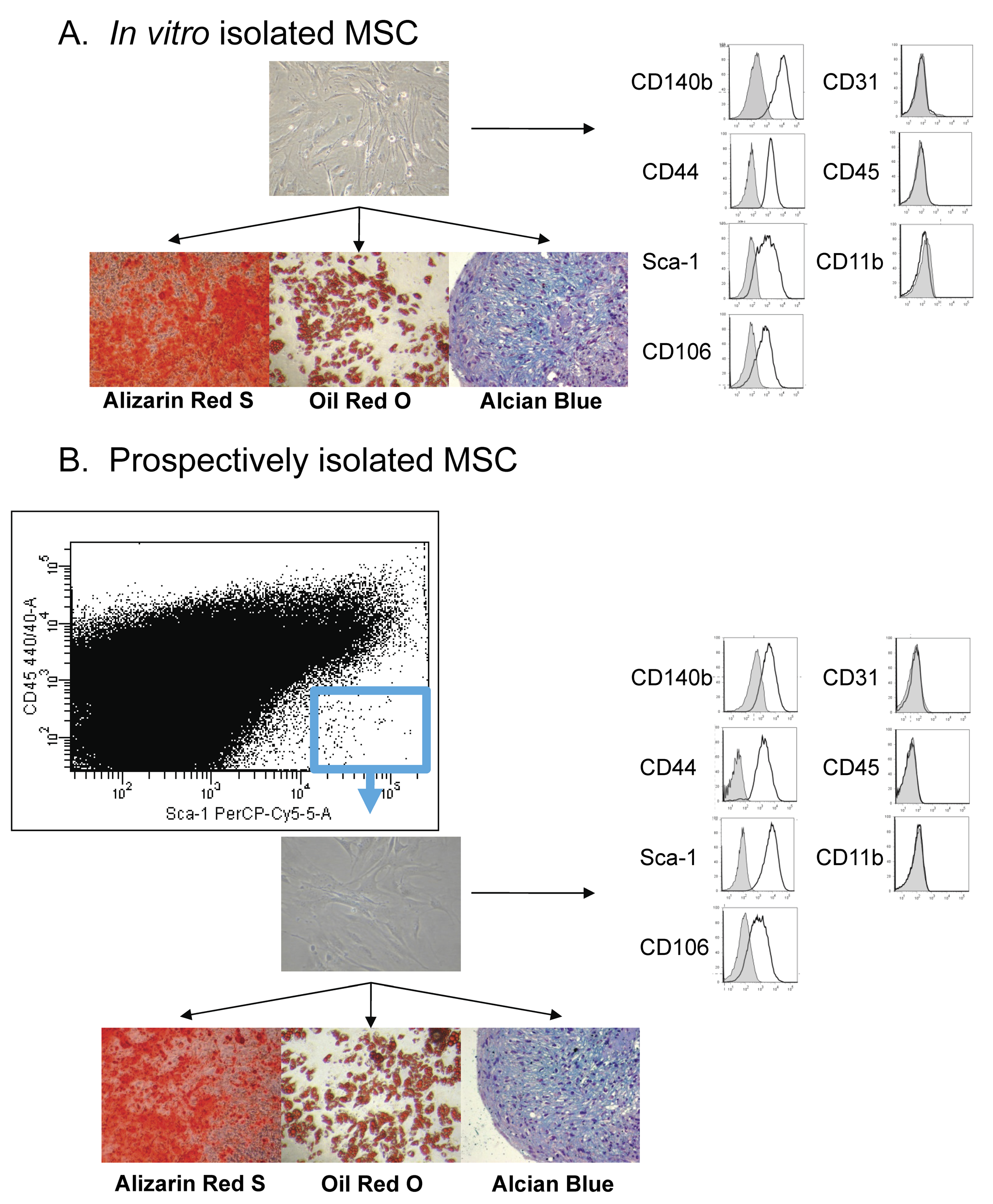

Supplement: Figure S3 — Characterization of MSC. mMSC were isolated by (A) in vitro plastic adherence or (B) prospective sorting of Lin− CD31− Sca-1+ cells. Cells from both populations were placed in culture and analyzed for bone, fat, and cartilage differentiation potentials as evidenced by Alizarin Red S, Oil Red O, and Alcian Blue staining, respectively. They were also phenotypically examined for CD44, Sca-1, CD140b, and CD106 expression and a lack of CD45, CD11b, and CD31. (TIF) [file pone.0030563.s003.tif]

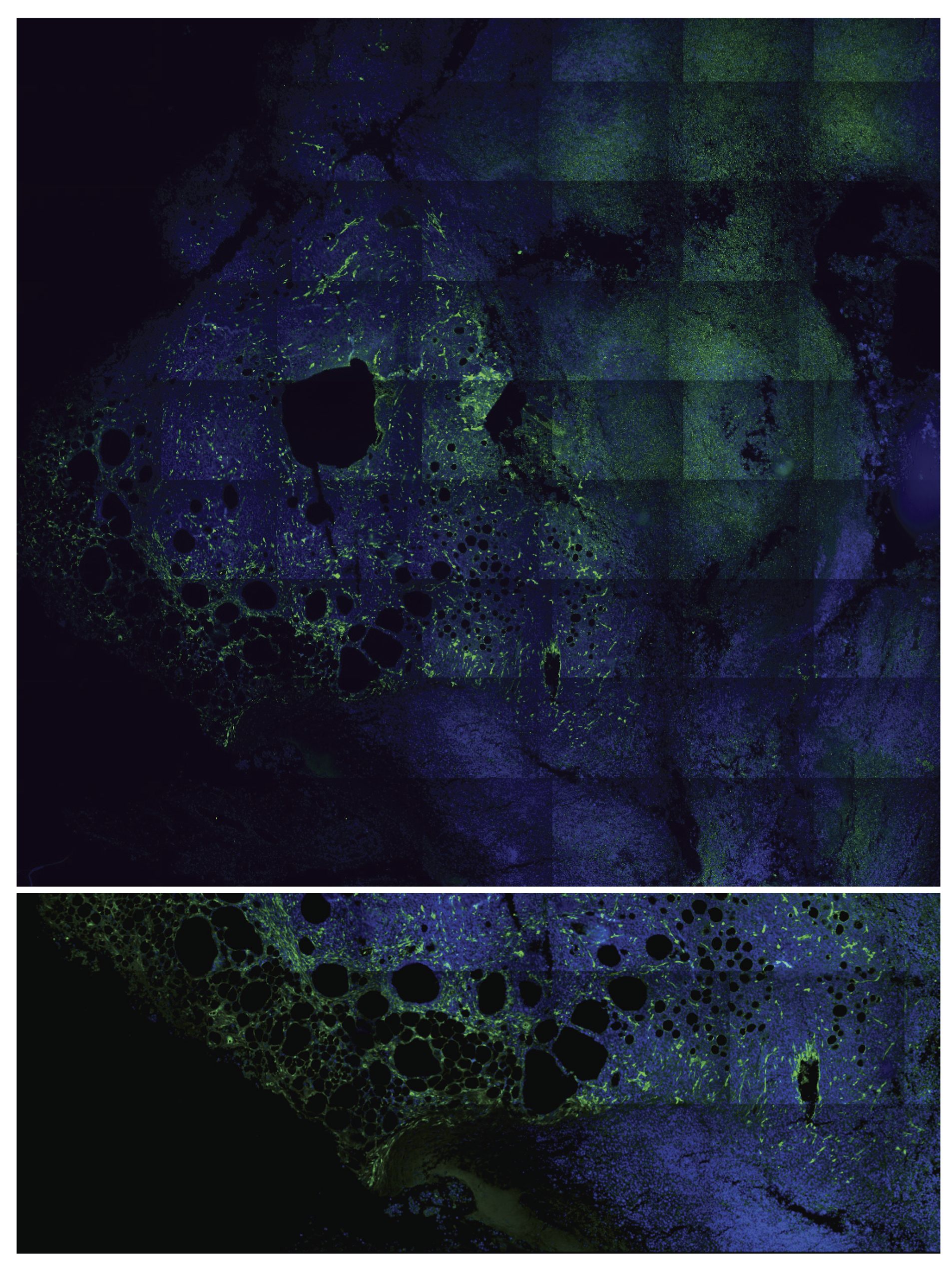

Supplement: Figure S4 — Engrafted GFP+ fat is locally recruited into the tumor microenvironment. GFP+ fat was subcutaneously implanted into wild type mice (n = 3). After engraftment, E0771 cells were subcutaneously injected. Two weeks later, sections of resected tumors and adjacent fat were analyzed for GFP expression. Analysis revealed engrafted fat with normal morphology. Recruited GFP+ fat derived cells remained in close proximity to the transplanted fat. (TIF) [file pone.0030563.s004.tif]
